# Whole-exome sequencing explored mechanism of selpercatinib resistance in *RET*-rearranged lung adenocarcinoma transformation into small-cell lung cancer: a case report

**DOI:** 10.1186/s12890-023-02799-5

**Published:** 2023-12-06

**Authors:** Yan Peng, Zhu Zheng, Wang Zewen, Liu Yanan, Zhang Mingyan, Sun Meili

**Affiliations:** 1 Oncology Department, Central Hospital Affiliated to Shandong First Medical University, Jinan, P. R. China; 2 Research Department, PLA Rocket Force Characteristic Medical Center, Beijing, P.R. China; 3 School of Medicine, Shandong University, Jinan, P. R. China

**Keywords:** NSCLC, *RET*-rearranged, Whole-exome sequencing, Small cell transformation

## Abstract

Small cell transformation was one mechanism by which *EGFR*-mutation NSCLC acquired resistance after tyrosine kinase inhibitors (TKIs) treatment. A few reports of small cell transformation occurred in other oncogene-driven lung cancers. We found the first case of transformation of a *RET*-rearranged lung adenocarcinoma to SCLC after selpercatinib, a novel highly selective *RET* TKIs. Whole-exome sequencing (WES) was used to explore alteration in gene expression in tumor tissue at initial diagnosis and after transformation into small cell carcinoma. We found that transformed into SCLC tumor tissue had inactivation of *RB1* and *TP53*, with *RET* fusion was still present. In addition, the *APOBEC* family of cytidine deaminases appeared amplification. Although *RET* rearrangement still existed, using another *RET* TKIs was ineffective, and etoposide plus platinum might be an effective rescue treatment.

**Supplementary Information:**

The online version contains supplementary material available at 10.1186/s12890-023-02799-5.

## Introduction

Genomic rearrangement of *RET* occurred in 1–2% of non-small cell lung cancer (NSCLC) and had been confirmed as an oncogenic driver in NSCLC [1]. Two highly selective *RET* tyrosine kinase inhibitors (TKIs), namely selpercatinib (LOX-292) and pralsetinib (Blue-667), had been approved by the FDA for *RET*-rearranged NSCLC. LIBRETTO-001 reported the median duration of response was 17.5 months with selpercatinib in *RET* fusion-positive NSCLC who had previously received platinum-based chemotherapy [2]. The ARROW study demonstrated that pralsetinib provided lasting benefits with 13.2 months in treatment-naive *RET* fusion-positive NSCLC and 16.4 months who had received platinum-based chemotherapy [3]. Despite the encouraging efficacy of highly selective *RET* TKIs, acquired resistance limited the duration of benefits.

Due to the low incidence of *RET*-rearranged in NSCLC and the late launch of highly selective *RET* TKIs, there were very few studies on acquired resistance of *RET* TKIs. Recent reports exploring mechanism of drug resistance to selective *RET* TKIs was driven by *RET*-independent resistance such as acquired *MET* or *KRAS* amplification [4]. At the end of 2022, two cases of small cell transformation following pralsetinib had been reported [5, 6]. But untill now, it has not been reported that another *RET* TKIs—selpercatinib can cause small cell transformation, and detailed mechanisms remain not been elaborated exhaustive.

We reported one case of transformation of a *RET-*rearranged lung adenocarcinoma to SCLC after selpercatinib treatment and explored its underlying mechanism using whole-exome sequencing (WES) technique.

## Case presentation

**Fig. 1:**
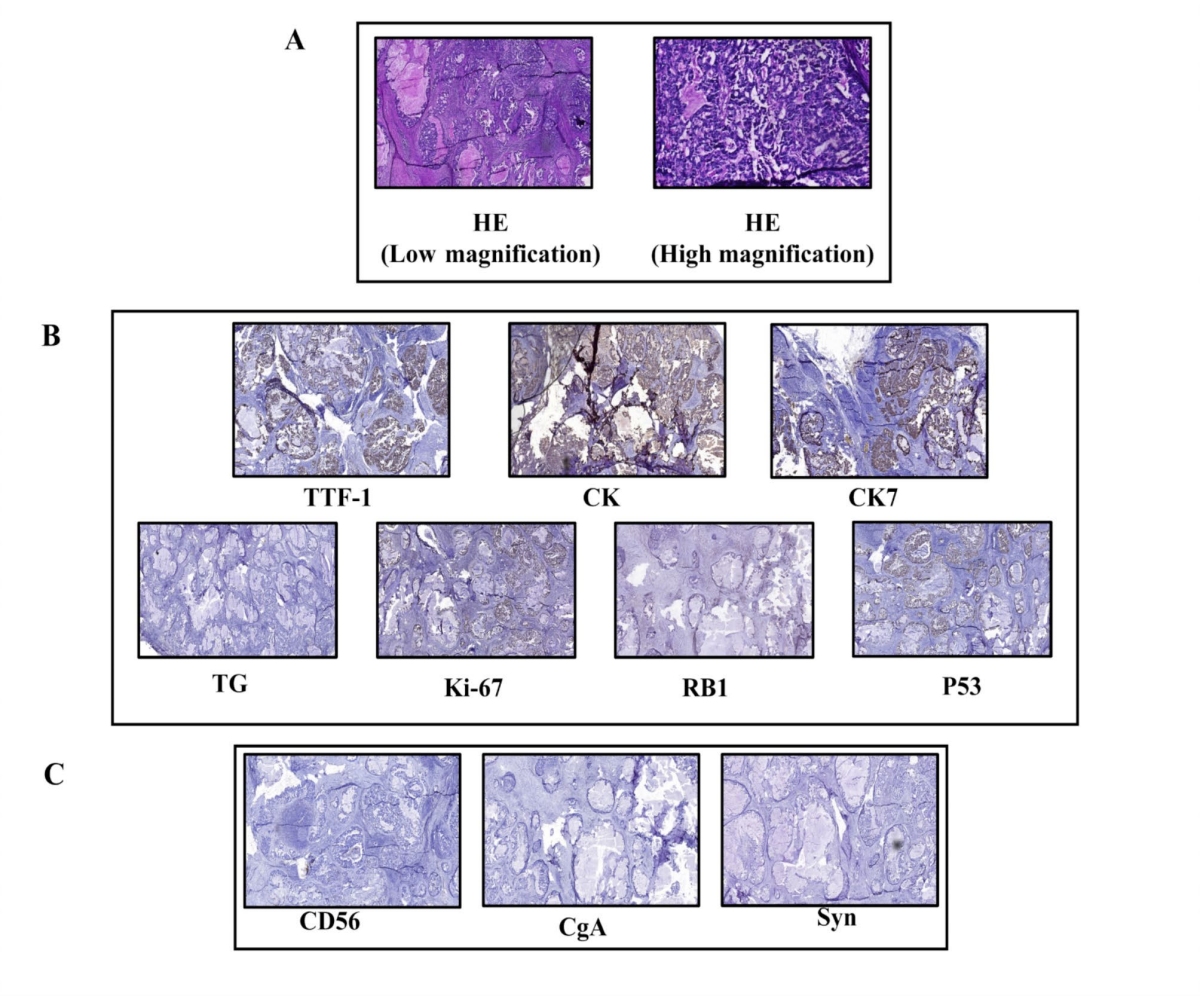
Hematoxylin and eosin (HE) and immunohistochemical (IHC) staining at initial diagnosis. (**A**) The HE staining results were in accordance with the morphological characteristics typically observed in adenocarcinoma. (**B**) The cervical lymph node biopsy immunohistochemistry showed TTF-1 (+), CK (+), CK7(+), which was diagnosed as lung adenocarcinoma, and TG (-) excluded thyroid cancer. P53 mutated, RB1 deletion and Ki-67 expression was 30%. (**C**) Neuroendocrine markers as CD56, CgA and Syn were all negative at initial diagnosis

**Fig. 2:**
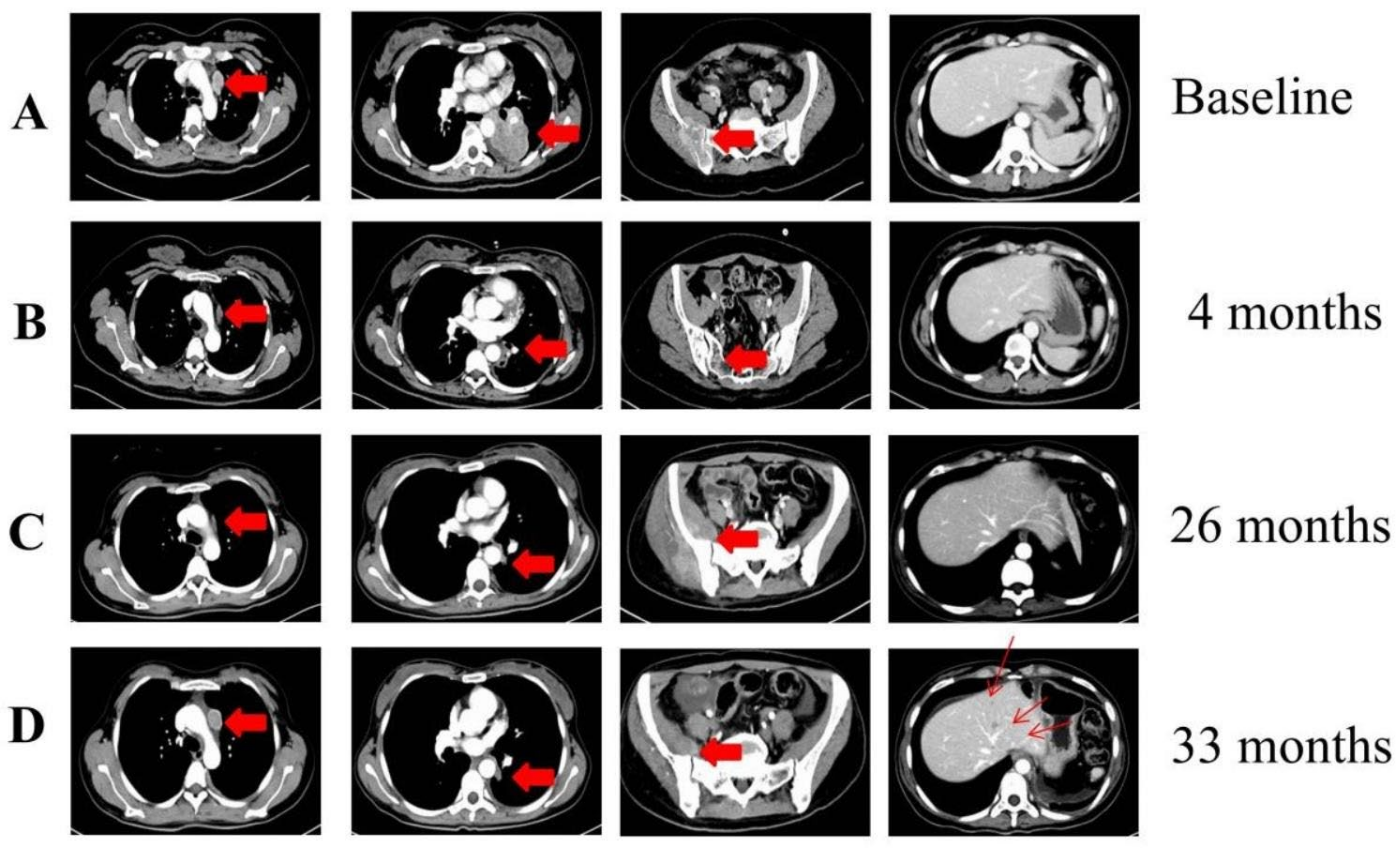
Imaging in diagnosis and treatment. An enhanced CT image of this patient at follow-up. (**A**) At initial diagnosis. Arrows indicated mediastinal lymph nodes, left lung masses, and soft tissue metastases of the right ilium, but no lesions in the liver. (**B**) After 4 months, the mediastinal lymph nodes, right lung, and right iliac crest lesions became significantly smaller and was assessed as PR. (**C**) After 26 months, the right iliac lesion was enlarged, but the rest of the lesions were not. Assessed as oligoprogression. (**D**) 33 months after taking selpercatinib, the mediastinal lymph nodes were enlarged and multiple intrahepatic metastases appeared

A 30-year-old Chinese woman who had never smoked was presented in December 2019 with a left neck mass. A biopsy revealed the histological characteristics of lung adenocarcinoma, immunohistochemical stains results showed:TTF-1(+), CK(+), CK7(+), TG(-), P53(+, mutation), RB1(-) and Ki-67(30%) (Fig. 1A&B). But the patient was not treated immediately because she was pregnant. In April 2020, genetic testing results showed that *KIF5B*-*RET* fusion. She agreed to participate in the LIBRETTO-001 study, and signed an informed consent form. She underwent radiographic examinations (craniocerebral and neck enhanced MRI and enhanced CT of the chest and abdomen) at baseline, shown in Fig. 2A. She was consistent with stage IVB (T2bN2M1c) by the American Joint Committee on Cancer eighth edition (AJCC 8th).

She started taking selpercatinib 160 mg twice a day from April 30th, 2020. After 4 months of treatment, the left lung lobe, mediastinal lymph nodes, and right iliac crest lesions were significantly receded, and the disease was assessed as partial remission (PR) (Fig. 2B). She experienced significant reduction in right hip pain. In terms of safety, there was no grade 3 or higher adverse events occurred (Common Terminology Criteria for Adverse Events (CTCAE) version 5.0). After 10 days of oral administration, she developed a grade 2 rash, which was reduced to grade 1 after symptomatic treatment. At the fifth month of oral administration, grade 2 liver function impairment appeared, which disappeared after liver conservation therapy. She had a small amount of pelvic effusion, which was assessed as a grade 1 adverse event. Unfortunately, 26 months after treatment, the patient’s right hip pain worsened again. CT showed significant enlargement of the right iliac metastases, although there was no progression of other target lesions (Fig. 2C). At this point, the disease was assessed as oligoprogression. Local radiotherapy for oligoprogression lesions was performed with a total dose of 56 Gy with fractionation dose of 2 Gy per time. Oral selpercatinib was administered without interruption.

**Fig. 3:**
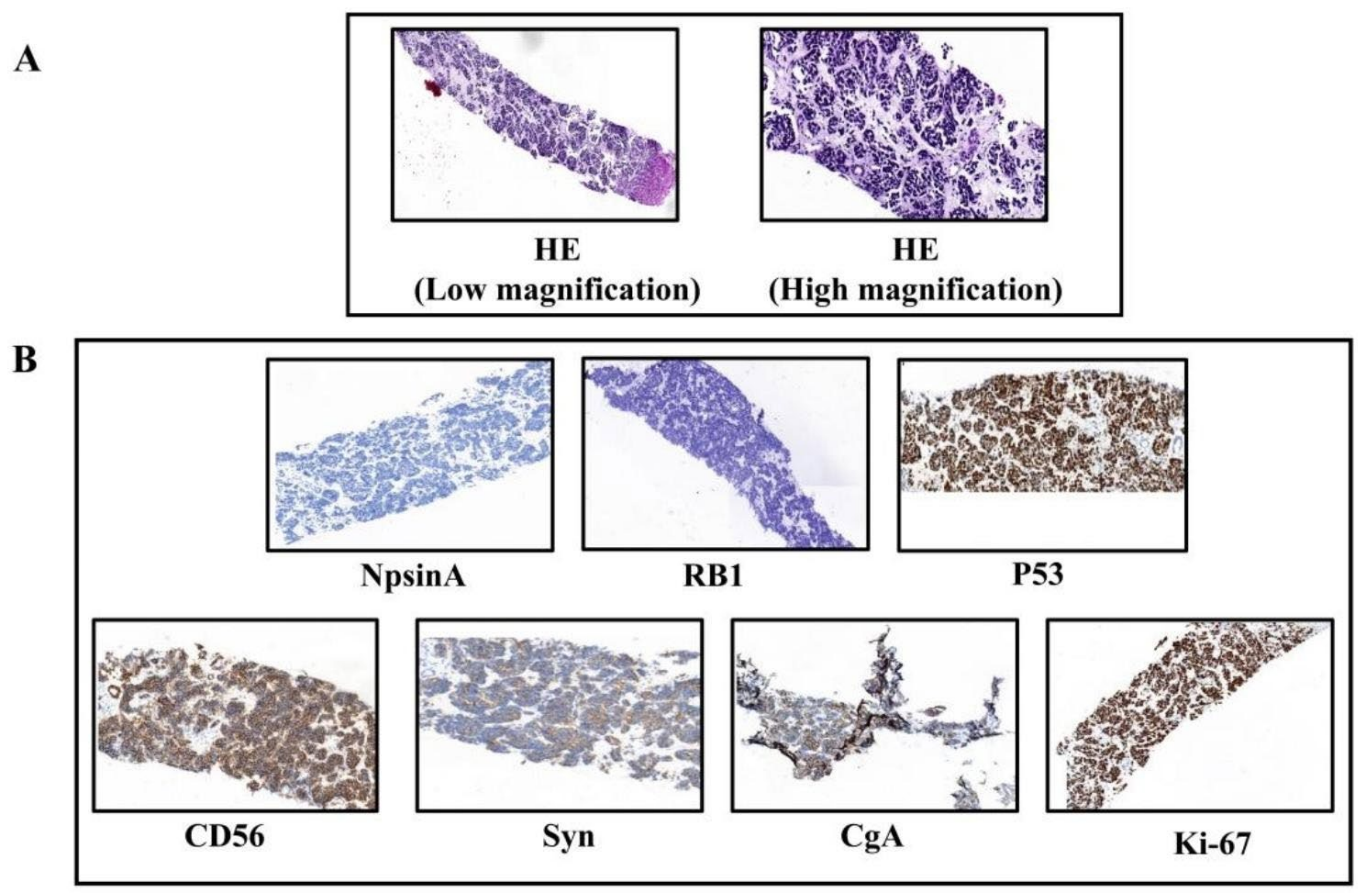
HE and IHC staining after liver metastasis. (**A**) HE staining showed that the cancer cells were small, the nuclei were dense and round or oval. (**B**) Adenocarcinoma marker NapsinA was negative, while neuroendocrine markers CD56, Syn and CgA were positive. P53 was mutant, RB1 was negative and Ki-67 expression was 80%

At the 33rd month of follow-up, the patient had mediastinal lymph node recurrent and multiple liver metastases (Fig. 2D). Progression disease (PD) was assessed with reference to Recist 1.1. We performed a liver metastatic biopsy, and the biopsy pathology revealed small cell carcinoma. HE staining and immunohistochemical staining were shown in Fig. 3, HE staining showed that the cancer cells were small, the nuclei were dense and round or oval (Fig. 3A), IHC showed NapsinA(-), RB1(-), P53(+, mutation), CD56(+), Syn(+), CgA(+), Ki-67(80%) (Fig. 3B). As a supplementary demonstration, the cervical lymph node tissue biopsied at the time of initial diagnosis was stained with neuroendocrine markers, but they were all negative (Fig. 1C), confirming that the small cell carcinoma was newly emerged.

After end of treatment (EOT) follow up, the patient was enrolled in a new Phase I study in March 14th 2023 with HS-10365, a novel *RET* TKIs, 160 mg orally twice a day. Reexamination after 1 month assessed that the disease continued to progress. At the end of April, the patient received chemotherapy with the etoposide/carboplatin (EC) regimen and had received 6 cycles of chemotherapy. The sixth cycle of chemotherapy was on August 10th, 2023, and the patient failed chemotherapy in August 31st due to new brain metastases.

With the permission of this patient, we applied whole-exome sequencing (WES) for tissues at initial diagnosis and after transformation into small cell lung cancer.

## Whole-exome sequencing approach

The DNA was fragmented into 180–280 bp fragments using the Covaris M220 crusher. The DNA library was prepared through processes including fragmentation, terminal repair, phosphorylation, and A-tailed addition. The capture reaction was conducted using capture Reagents (Nanodigmbio) according to the manufacturer’s instructions. The capture reaction probes were synthesized by Integrated DNA Technologies (IDT, Coralville, IA). The libraries, each with a specific index, were hybridized with a liquid phase containing up to 543,872 biotin-labeled probes. Streptomycin beads were then utilized to capture 334,378 exons from 20,965 genes.

The library that was constructed underwent quantification using Qubit 4.0, while the evaluation of DNA quality and fragment size of the libraries was conducted using the High Sensitivity D1000 Kit for TapeStation 4150 (Agilent, CA, USA). The libraries that met the necessary criteria were then subjected to sequencing using PE100 on the DNBSEQ-T7 platform (MGI, Shenzhen, China). PE100 denoted a high-throughput double-ended sequencing method, where 100 bp was measured at each end.

The fastq files were subjected to quality filtering using fastp (0.23.2). The Burrows-Wheeler Aligner (BWA-0.7.12) was employed for aligning the sequences to the human reference hg19. Subsequently, SAMtools (version 1.3) was utilized for the conversion, sorting, and indexing of the alignment data. To mitigate biases, the duplicates were identified and marked using SAMBLASTER (Version 0.1.22).

VarDict (1.7.0) were used to identify somatic mutations, including single nucleotide variants (SNVs) and insertions and deletions (indels). Ensembl Variant Effect Predictor (V97) was used to annotate the mutations.

The Delly software (v0.8.72) was employed for gene fusion detection. These algorithms heavily depend on breakpoints to accurately determine genome rearrangements at the single-nucleotide level. Variant Allele Frequencies (VAFs) was calculated as RV/(RR + RV), RR stands for “high-quality reference junction reads”, and RV stands for “high-quality variant junction reads”.

Tumor mutational burden (TMB) was defined as the number of somatic synonymous mutations per megabase in each sample, with hotspot/fusion mutations excluded. MSIsensor (v0.2) was used to detect microsatellite instability (MSI) status. Then, the MSI value was recalculated and corrected using the in-house tool.

CNVnator software (version 0.3.2) was employed for the identification of copy variation. The genome was partitioned into non-overlapping windows of equal length, and the number of matched reads within each window was normalized as a depth signal to facilitate the detection of copy number variation. The normalization process involved standardizing the number of matched reads in each window. A window length of 100 bp was selected as per standard parameters and settings. Subsequently, the detected CNV results were annotated using ANNOVAR software available at http://www.openbioinformatics.org/annovar/.

## Whole-exome sequencing results

### Somatic mutations

**Fig. 4:**
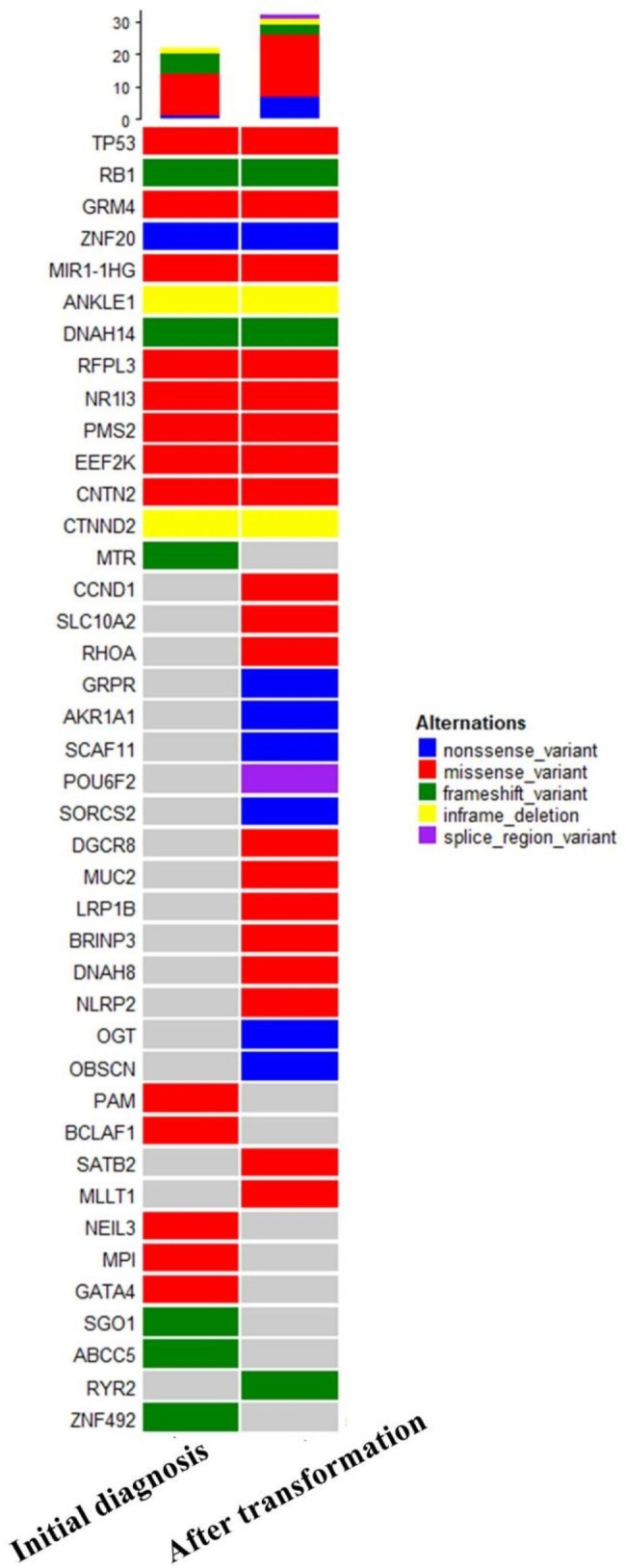
A heatmap of significant genetic events at initial diagnosis and after transformation. Legend shown missense variants, nonsense variants, frame-shift variants, in-frame deletions, splice variants in different color. Gray indicated that no mutation occurred

A total of 37 somatic mutations were detected in initial diagnosis biopsy specimens, including 21 missense variants, 3 nonsense variants, 6 frame-shifting deletions, 4 frame-shifting insertions, 2 in-frame deletions and 1 splice region variants. After transforming into SCLC, 74 somatic mutations were detected, including 50 missense variants, 10 nonsense variants, 1 synonymous variants, 4 frame-shifting deletions, 2 frame-shifting insertions, 3 in-frame deletions, 3 splice region variants and 1 splice site variants. After enriching tumor-related signaling pathways using database for annotation, visualization, and integrated discovery (DAVID) enrichment analysis, genes were screened and presented in heat map (Fig. 4). 13 gene mutations were present initial diagnosis and after transformation, 19 gene mutations were emerging after transformation, and 9 gene mutations were disappeared after transformation.

### Gene fusions

**Table 1:** Gene fusions at initial diagnosis and after transformation

Initial diagnosis (Adenocarcinoma)			After transformation (SCLC)		
Chromosome	Genetic fusion	Reads/VAF	Chromosome	Genetic fusion	Reads/VAF
Chr10	*KIF5B*》*RET*	0.3922	Chr10	*KIF5B*》*RET*	0.3965

The fusion genes initially diagnosed in this patient was *KIF5B-RET* fusion with the abundance of 39.22%. After transformation into small cell lung cancer, *KIF5B-RET* was still present with a abundance of 39.65%. No other meaningful fusions newly emerged (Table 1).

### Copy number variation (CNVs)

**Fig. 5:**
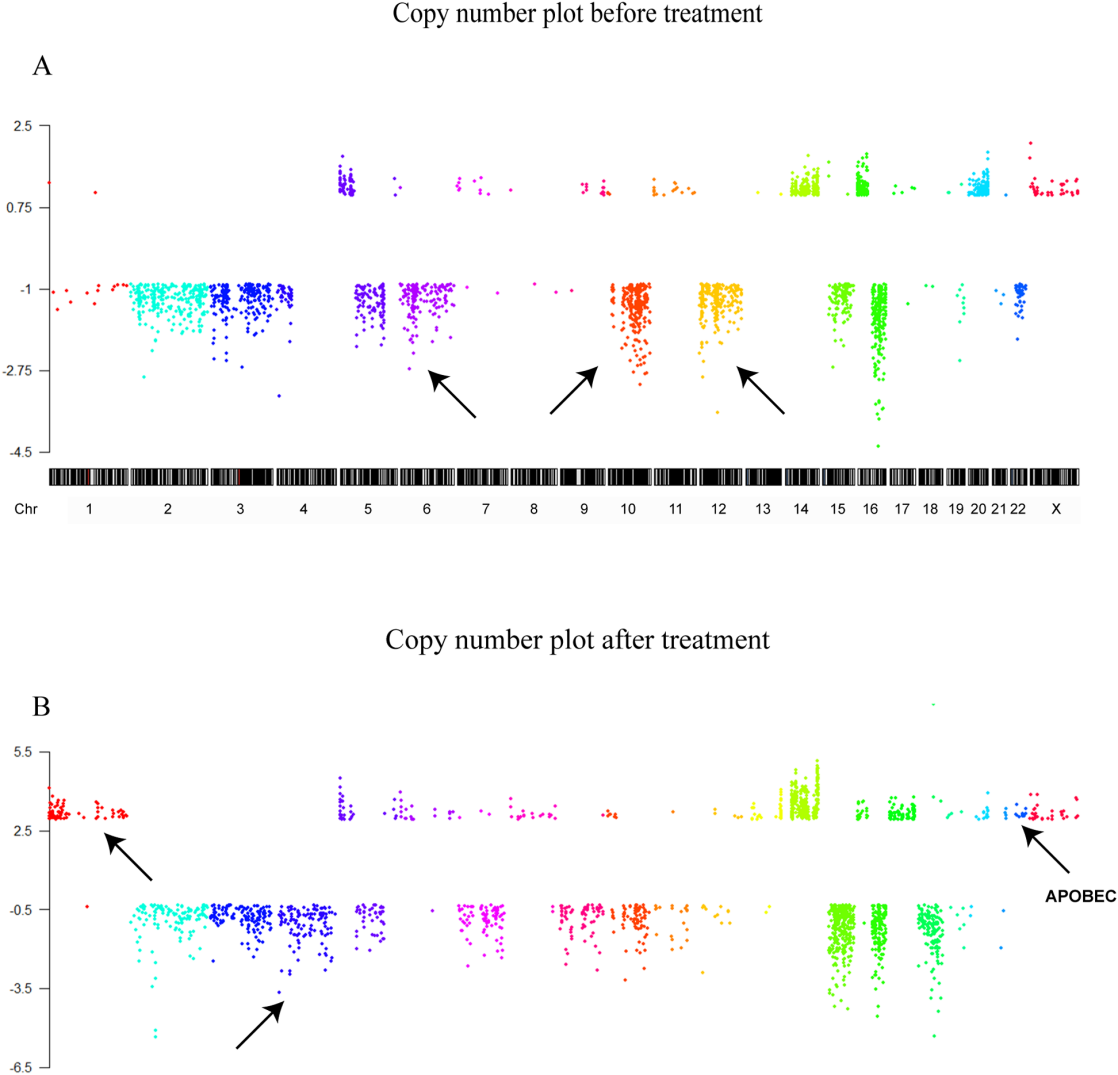
Copy number variations at initial diagnosis and after transformation. The X-axis was the sequence of Chromosomes, the Y-axis was the fold change of variation, and the arrows indicated the locations of Chromosomes with large copy number variation at initial diagnosis and after transformation into SCLC

As shown in Fig. 5, after transformation into SCLC, CNVs profile showed gain of Chromosome 1 and Chromosome 22q, while loss of Chromosome 4. The CNVs gain of *APOBEC-3 F* and *APOBEC-3G* were detected in Chromosome 22q13.1 after transformation to SCLC, while this situation was not observed at initial diagnosis.

### Mutational signatures

**Fig. 6:**
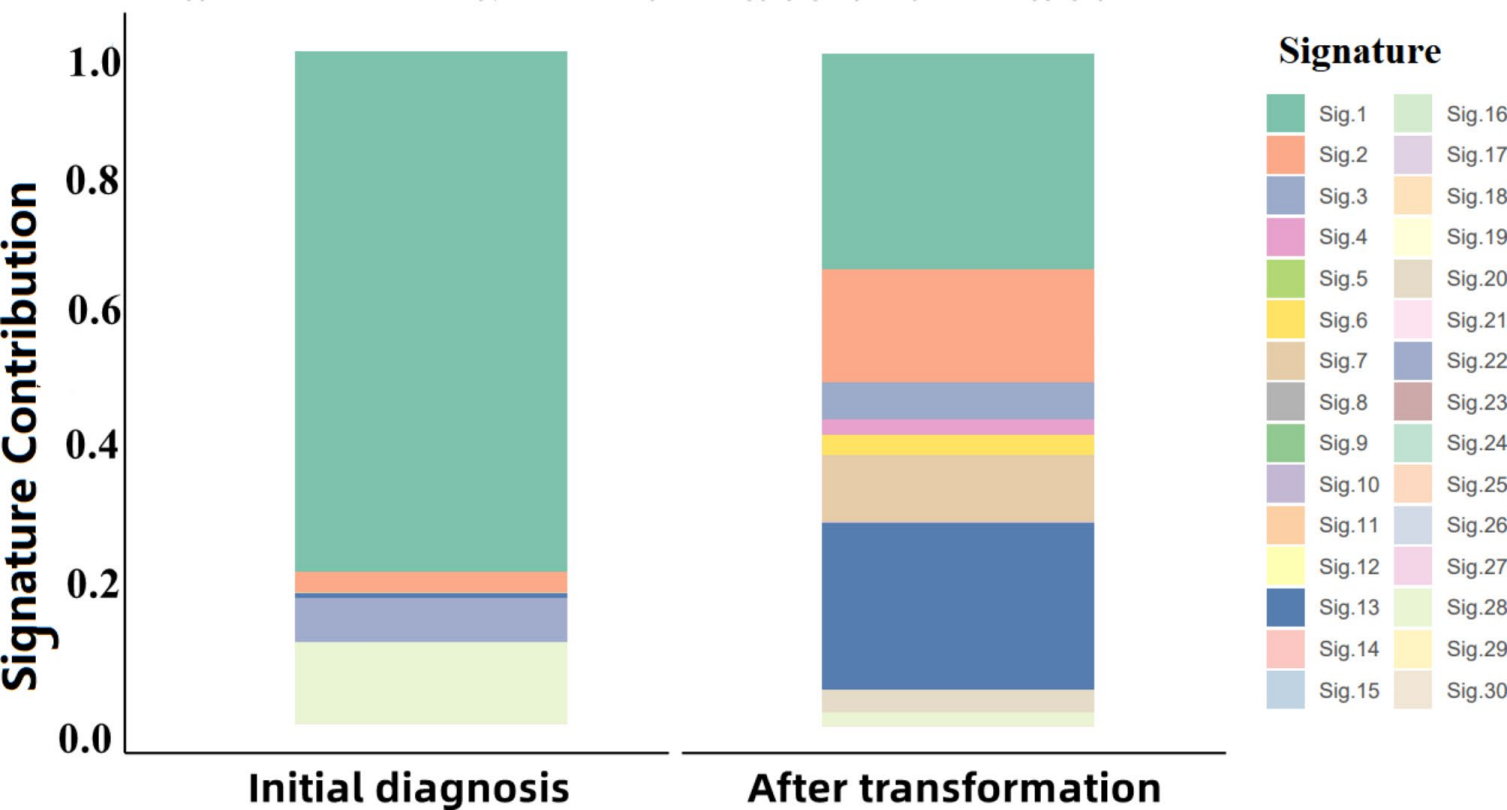
Mutational Signatures at initial diagnosis and after transformation. Each individual mutation signature represents a different proportion of six types of base substitutions (C > A, C > G, C > T, T > A, T > C, T > G). Mutation signatures are characterized predominantly by C > T (signatures 1 A/B, 6, 7, 11, 15,19), C > A (4, 8, 18), T > C (5, 12, 16, 21) and T > G mutations (9, 17), and others showing distinctive combinations of mutation classes (2, 13, 14). The X-axis represented the samples at initial diagnosis and after transformation, and the Y-axis represented mutation frequency. Different mutational signatures were represented by different colors, as indicated in the legend

The proportion of signature 2 and signature 13 increased significantly after transformation to SCLC compared to initial diagnosis. Signature 2 had a higher frequency of C > T mutations whereas signature 13 had a higher frequency of C > G mutations (Fig. 6).

### Cancer biomarkers

**Table 2:** Cancer biomarkers at initial diagnosis and after transformation

	TMB	TNB	MSI-Score
Initial diagnosis (Adenocarcinoma)	1.10	1.39	0.25
After transformation (SCLC)	2.21	1.01	1.16

As shown in the Table 2, the TMB was 1.10 at the initial diagnosis and 2.21 after SCLC transformation, both of which were less than 2.5, indicating that the TMB-low. TNB (predicted number of neonatal antigens/size of coding region) was 0.39 at initial diagnosis, 1.01 after SCLC transformation, and TNB-low (TNB < 0.5 Muts/Mb) at initial diagnosis, while TNB-middle (0.5 Muts/Mb ≤ TNB ≤ 4.5 Muts/Mb) after transformation. Intratumor heterogeneity (ITH) index, number of subclonal mutations /(master clone + number of subclonal mutations), 0.86 at initial diagnosis, 0.55 after transformation. The microsatellite (MSI) score was 1.25 at initial diagnosis and 1.16 after conversion, both of which were MSI-low.

## Discussion

We observed a small cell transformation after selpercatinib in a female NSCLC patient with *RET-*rearrangement. To explore underlying mechanism, we performed WES at initial diagnosis and after transformation samples. It was found that some somatic mutations disappeared, some somatic mutations newly arising and some always presence before and after transformation. In always presence genes, we observed that dual inactivation of the tumour suppressor genes *RB1*(c.240dup) and *TP53* (c.844 C > T). *RB1* and *TP53* inactivation had been reported a marker for transformation into small cell lung cancer in *EGFR* mutation [7], *ROS1* fusion [8] and *ALK* rearranged [9] lung adenocarcinoma. In NSCLC with *EGFR* mutation, the loss-of-function of *RB1* and *TP53* increased the 43 folds greater risk of SCLC transformation [7]. It was also found that the both alterations of these two genes resulted in a higher SCLC transformation rate than a single gene mutation [10]. *RB1* loss can also enhance SCLC transformation by upregulating epigenetic and stem cell reprogramming factors [11]. Furthermore, evidence has shown that *TP53* loss could effectively promote the transformation of alveolar type II cells, suggesting that *TP53* can enhance SCLC transformation [12]. The small cell transformation population, irrespective of the oncogene-driven factor, was determined to be associated with *RB1* loss and *TP53* mutation in NSCLC. In addition, the expression of Ki-67 after transformation into small cell was significantly up to 80%, which was also a significant finding in this case.

*KIF5B*-*RET* fusion was still detected in tissues after small cell transformation, and there was no significant change in mutation abundance. The persistence of fusion lead us to believe strongly that NSCLC transformed into SCLC in this case rather than histologically NSCLC mixed SCLC component. Considering that *RET*-rearrangement still existed, another *RET* TKIs was considered, but there was no effective response.

There was a difference in copy number variations at initial diagnosis and after small cell transformation, in which the most noteworthy were the copy number amplification of *APOBEC-3 F* and *APOBEC-3G*. In human cells, APOBEC proteins could mutate single-stranded chromosomal DNA resulting in high levels of mutations in multiple tumor types [13, 14]. It has also been reported that tyrosine kinase inhibitors (TKI) could induce APOBEC-3 A in NSCLC, which promoted the emergence of drug-tolerant persister clones. APOBEC3A was activated by NF-κB1 and induced DNA damage to promote resistance to targeted therapies [15]. The current analysis could provide further evidence that the APOBEC-induced mutational process can be hyperactivated during transformation into SCLC [7]. After transformation into small cell lung cancer, mutational signatures 2 and 13 contribution were significantly increased. Signature 2 and 13 were characterized primarily by C > T and C > G mutations at TpCpN trinucleotides [16]. Signature 2 has a higher proportion of C > T substituted, and signature 13 has a higher proportion of C > G substituted. This two signatures have been attributed to the APOBEC family of cytidine deaminases [16, 17]. This corroborated that the APOBEC family lead to the change of mutation signatures and played a key role in the transformation.

The TMB after transformation was 2.21, slightly higher than initial diagnosis, and was microsatellite stable, suggesting poor immune efficacy after transformation. A previous study indicated that transformed SCLC tumors did not respond to immunotherapy [18]. The patient demonstrated a progression-free survival duration of 4.2 months when treated with the EC regimen. In another case report [6], after *RET* TKIs resistance and transformed into SCLC, treated with etoposide-carboplatin chemotherapy had achieved partial response after 6 cycles. Therefore, for *RET-*rearranged cases transformed into SCLC, it might be less benefit to accept another *RET* TKIs, and etoposide plus platinum might be an effective rescue treatment. Whether TKIs did not interrupt combination with the chemotherapy could got better response, there was no evidence until now.

There were compelling justifications supporting the notion that SCLC originated from NSCLC transformation rather than mixed SCLC and NSCLC. Firstly, the identification of identical clones and subclones in tissues obtained prior to treatment and those obtained subsequent to transformation provided substantial evidence (Supplementary 1). Secondly, the persistence of the *RET* fusion state in SCLC following transformation, with comparable levels of abundance, further supports this hypothesis. Lastly, the extended progression-free survival observed in NSCLC aligns with its biological behavior, in contrast to SCLC. We maintain a strong conviction regarding the shared origins of the pre- and post-transformed tissues.

Recently, SCLC had been classified into four molecular subtypes based on the relative expression of four transcriptional regulators, *ASCL1*, *NEUROD1*, *POU2F3*, and *YAP1* [19]. Each of these molecular subtypes showed distinct therapeutic vulnerabilities. These four transcriptional regulators might possess potential significance in the process of small cell transformation. Regrettably, there was an insufficient amount of tissues remaining for the identification of these four transcription factors.

In conclusion, our study showed that *TP53* and *RB1* inactivation were common in SCLC transformation regardless of any oncogene-driven NSCLC. Tissue biopsy and Next Generation Sequencing (NGS) were particularly important for diagnosis when disease progression. Histological transformation into small cell lung cancer was a new mechanism of acquired resistance to *RET* TKIs. APOBEC induced hypermutation might be one of the mechanisms of small cell transformation. Although *RET*-rearrangement still existed, using another *RET* TKIs was ineffective, and etoposide plus platinum might be an effective rescue treatment.

### Electronic supplementary material

Below is the link to the electronic supplementary material.

**Supplementary Material 1:** Overlap of subclone evolution before and after transformation

**Supplementary Material 2:** Gene fusion detection

## Data Availability

All data generated or analysed during this study are included in this published article.
